# Sézary Syndrome Biomarker, T Cell Transcription Factors and Cytokine Genes Provide Novel Insight into Response During Mogamulizumab Treatment

**DOI:** 10.3390/cancers18142304

**Published:** 2026-07-17

**Authors:** Alanna Davis, Jun Ying, Ping-Ching Hsu, Jeffrey Chen, Khiem Tran, Henry K. Wong

**Affiliations:** 1 College of Medicine, University of Arkansas for Medical Sciences, Little Rock, AR 72212, USA; adavis52@northwell.edu; 2 Department of Biostatistics, University of Arkansas for Medical Sciences, Little Rock, AR 72205, USA; jying@uams.edu; 3 Department of Environmental Health Sciences, University of Arkansas for Medical Sciences, Little Rock, AR 72205, USA; phsu@uams.edu; 4 Department of Dermatology, University of California, 3350 La Jolla Village Dr., San Diego, CA 92093, USA; jhc128@health.ucsd.edu; 5 Department of Dermatology, University of Arkansas for Medical Sciences, Little Rock, AR 72212, USA; 6 Dermatology Service, Veterans Administration San Diego Health System, San Diego, CA 92161, USA

**Keywords:** Sezary syndrome, T cell lymphoma, biomarker, gene expression, therapy, mogamulizumab

## Abstract

***Background***: Novel Sézary syndrome (SS) biomarker genes identified previously through transcription profiling were examined for changes in expression after mogamulizumab treatment. Incorporating new biomarkers for measuring response to disease would improve clinical care. ***Objective***: To assess novel SS biomarker and cytokine genes in patients treated with mogamulizumab with clinical response, blood response, and immunologic parameters. ***Methods***: We performed a real-world case–control and case–case study analyzing the expression of SS biomarker genes in peripheral blood mononuclear cells (PBMCs) from six SS patients treated with mogamulizumab using qRT-PCR. ***Results***: We demonstrated a reduced expression of SS biomarker genes in PBMCs of SS patients following mogamulizumab therapy. In our cohort, five of the SS biomarker genes (*PLS3*, *TWIST1*, *KCNK1*, *DNM3* and *TOX*) showed statistically consistent high expression compared to normal PBMCs at baseline. After treatment with mogamalizumab, these five SS biomarkers showed significant correlations with skin response (*p* < 0.05) and three (*TOX*, *TCRL3* and *DNM3*) with blood response (*p* < 0.05). A decrease in *GATA3* expression correlated to an improvement in skin severity, and *STAT4* inversely correlated to Sézary cell decrease (*p* < 0.05). Two cytokine genes, *IL4* and *IFNG*, reflective of an immune response that is abnormal in SS, showed a trend to normalized expression with *IL-4* decreasing and *IFNG* increasing after treatment. ***Limitations***: This was a real-world study of patients who failed prior treatments, with a small sample size and variable timing between patient sample collection. ***Conclusions***: Unique SS biomarker, cytokine and T cell transcription factor genes are valuable in assessing molecular and immune responses following treatment.

## Simple Summary

Sézary syndrome is a rare and aggressive cancer of T cells that affects the skin and blood. The disease can be difficult to diagnose and monitor with standard clinical tests. This translational study demonstrates the utility of novel SS biomarker genes previously identified to be differentially expressed between normal T cells and Sezary T cells. In a real-world study, we report that treatment of SS patients with a monoclonal antibody, mogamulizumab, leads to a decrease in novel SS biomarker gene expression as well as a trend to normalization of T cell transcription factors and cytokine genes. These measurable changes in expression were significant and correlated with disease severity and response efficacy. These results support that these previously identified SS biomarker genes could be useful as markers of Sézary syndrome and could offer clinicians a more precise and sensitive approach to evaluate treatment response, detect residual disease, and assess immune parameters.

## 1. Introduction

Cutaneous T cell lymphoma (CTCL) is a group of rare lymphoproliferative diseases driven by the heterogeneous proliferation of abnormal skin homing lymphocytes. Sézary syndrome (SS) represents less than five percent of CTCL cases and is an aggressive CD4 leukemic variant, characterized by erythroderma, lymphadenopathy, severe pruritus, and circulating malignant cells in the peripheral blood [1,2,3].

Diagnostic challenges arise when SS resembles benign inflammatory dermatitis, prompting studies on the identification of molecular biomarkers to improve the accuracy of diagnosis, and monitoring response to treatment [4,5]. Typically, monitoring progression and/or treatment efficacy in SS involves flow cytometry, but more specific biomarkers that correlate with skin clinical response have not been extensively studied [5]. Moreover, there remain limitations with respect to data interpretation as well as correlating those results with clinical response or disease progression. Addressing this continual need, we and others have previously identified novel upregulated biomarker genes for SS from gene expression profiling of peripheral T cells isolated from SS patients [6,7,8,9,10]. The role of these genes in the development and progression of SS is unclear, but several have been implicated in cancer metastasis, T cell exhaustion, and autoimmunity [11,12,13,14].

Therapeutic options for SS have increased, most recently with agents targeting specific surface molecules [15,16,17,18]. One FDA-approved targeted therapy for CTCL is mogamulizumab, a monoclonal antibody against CCR4, a cell surface chemokine receptor necessary in lymphocyte trafficking [19,20]. In a phase 3 trial, CTCL patients showed clinical responses to mogamulizumab, with a higher response in the blood compared to the skin [19,20].

Although flow cytometry is used to monitor blood involvement in Sezary syndrome, the identification of unique genes highly expressed in Sezary syndrome offers new additional methods to monitor disease response [21,22]. In this study we focus on nine SS biomarker genes (*PLS3*, *TWIST1*, *KCNK1*, *DNM3*, *TOX*, *NEDD4L*, *GNG4*, *FCRL3*, and *TIGIT*) that show profound high abnormal expression in SS from prior studies and measured their changes in expression post treatment [8,9]. We show that the expression of these SS genes in peripheral blood decreased but the level approached normal following treatment with mogamulizumab. Our results support their use as diagnostic biomarkers in monitoring treatment and clinical response with mogamulizumab in SS. We also analyzed cytokine gene expression in SS patients, and show that immune balance is disrupted, which increases the risk of severe infections and death. We demonstrate that upon treatment with mogamulizumab, both T cell transcription factors and cytokines trend to normal, favoring Th1 polarization.

## 2. Materials and Methods

### 2.1. Patients

The study received IRB approval status: UAMS IRB Protocol #204503; VA #1443158. Informed consent was obtained from all subjects involved in the study. Subjects were at least 18 years of age and qualified for inclusion by disease criteria according to the WHO classification of cutaneous lymphomas [22]. All subjects had erythroderma, a highly elevated CD4/CD8 ratio, and/or atypical circulating Sézary cells determined by flow cytometry (Table 1). All SS patients received at least four prior systemic treatments and had progressive disease prior to treatment with mogamulizumab. SS subjects provided blood specimens before initiation of mogamulizumab and post mogamulizumab for analysis. As this is a real-world study, procurement of post-treatment blood specimens was variable based on schedules that were agreeable for providing additional blood for research, but was all within 60 days post initiation. We performed clinical assessments at each visit associated with blood collection. Healthy anonymous donor samples were obtained from the blood bank and included if they had no significant medical diagnoses.

### 2.2. Cells and Treatments

Peripheral blood mononuclear cells (PBMCs) were isolated from Sézary patients and normal healthy donors (HDs) using Ficoll-Paque density gradient medium (Mediatech, Inc., Manassas, VA, USA) centrifugation. Isolated PBMCs were then stimulated with phorbol myristate acetate (PMA) at 25 ng/mL and A23187 calcium ionophore at 0.1 µg/mL and cultured for 2 and 6 h in RPMI media followed by RNA isolation to measure kinetic changes in gene expression for cytokine genes, as described previously [23,24].

### 2.3. Quantitative Real-Time PCR (qRT-PCR) Analysis

Total RNA from PBMCs was purified, reverse transcribed, and subjected to qRT-PCR as described previously [23,24]. Each assay included a negative control (no template DNA), and reactions were performed in triplicate using Maxima SYBR Green qPCR master mixes (Life Technologies, Carlsbad, CA, USA), on a QuantStudio 5 Real-Time PCR system. B2-microglobulin mRNA expression was used for normalization of CT values. Primer sequences are provided in Supplementary Table S1.

### 2.4. Data Analysis and Statistical Evaluation

Relative quantification of gene expression between SS and HDs employed the 2^−ΔΔCT^ method normalized to expression of HDs [24]. Statistical analysis was performed with GraphPad Prism 9.2.0 software and JMP Version 16.2.0. A comparison of gene expression between the two groups was performed via the Mann–Whitney U-test after adjusting for multiple comparisons using the Benjamini–Hochberg procedure. *p*-values comparing pre-treatment and post-treatment gene expression were calculated using the Wilcoxon signed-rank test. To determine the diagnostic value of the biomarker genes for classifying SS, we analyzed qRT-PCR expression data from SS and HD PBMCs using the receiver operating characteristic (ROC) methodology performed in GraphPad Prism 9.2.0. The (ROC) area under the curve (AUC) is a measure of the quality of a model, that is, a measure of the association of predicted probabilities and observed responses. An AUC value close to 1 indicates a better diagnostic test while values approaching 0.5 indicate that a diagnostic test is no better than random chance.

### 2.5. Clinical Response Evaluation

Baseline demographics were compared across the groups in a post hoc analysis using simple *t*-tests. Clinical response was evaluated in SS patients in the skin and blood through skin and blood response scores [22]. A skin score was derived through multiplication of body surface involvement and level of erythema determined by a 0–4-point severity scale. Blood response was evaluated using a CD4^+^CD26^−^ population using flow cytometry (Table 2).

Clinical response scores of the skin and blood were correlated with log2 transformed mean fold-change expression levels of each of the eleven biomarker genes through a linear mixed effect model with repeated measures (Table 3).

## 3. Results

### 3.1. Patient Population

This study incorporated a case–control design comparing novel biomarker gene expression between SS patients and HDs in a real-world study. We also incorporated a case–case design comparing changes in gene expression at baseline and after treatment with mogamulizumab. We based our selected differentially expressed genes in SS T cells on Moerman-Herzog [8]. Six SS patients who met SS disease criteria and 11 healthy subjects participated. The demographics among the SS patients and HDs did not show significant differences among age and sex (Table 1). One patient discontinued treatment due to an adverse reaction (rash). The median duration of mogamulizumab treatment was 11 months. The clinical responses for change in body surface area, erythema and Sezary count at baseline prior to treatment and post treatment are reported (Table 2).

The clinical responses for subjects with treatment are shown in (Table 2). Subject 3 and 6 showed complete cutaneous responses; other subjects showed a partial response.

### 3.2. SS Biomarker Gene Analysis

**Figure 1:**
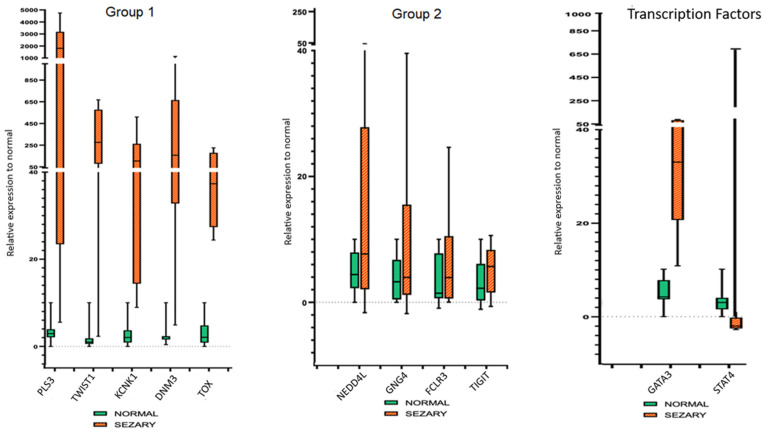
Expression level separate groups of SS biomarker gene expression in PBMCs compared to expression in HDs. Group 1 consists of SS biomarker gene levels that are significantly higher than HD. Group 2 displays biomarker genes showing intermediate levels of differential expression in SS when compared to biomarker expression levels in HDs. Transcription factors *GATA3* and *STAT4* show differential expression in PBMCs from SS compared to HDs.

Nine biomarker genes highly differentially expressed in SS compared to non-SS patients were selected for analysis using qRT-PCR (*PLS3*, *TWIST1*, *KCNK1*, *DNM3*, *TOX*, *NEDD4L*, *GNG4*, *FCRL3*, and *TIGIT*) (Figure 1) [7]. T helper transcription factor genes, Th2(*GATA3*) and Th1(*STAT4*), and cytokine genes (*IFNG*, *IL2*, *IL10*, *IL17A*, *IL4* and *IL13*) were included to monitor immune bias changes after treatment [25]. The results from different SS biomarker genes based on the degree of increased expression compared to HDs at baseline (Figure 1) are shown in this SS cohort using qRT-PCR. Prior to mogamulizumab treatment, the expression of all genes, except for *STAT4*, showed higher mean expression in SS PBMCs compared to HDs, with the expression level of five of these genes (*PLS3*, *TWIST1*, *KSNK1*, *TOX* and *DNM3*) being statistically significant (Table 3).

Based on the expression level of the biomarker genes for SS and HDs, we determined ROC curves to assess the ability of these genes to classify SS accurately (Table 4). Good discrimination capacity for sensitivity and specificity was seen for all 11 genes analyzed. Out of these 11 genes, six (*TOX*, *GATA3*, *PLS3*, *DNM3*, *TWIST1*, and *KCNK1*) showed an AUC value (maximum 1) above 0.9 and both sensitivity and specificity above 80%, denoting them as potential valuable classifiers for distinguishing SS from non-SS samples (Table 4).

### 3.3. Changes in Biomarker Genes in SS PBMCs Post Treatment

**Figure 2:**
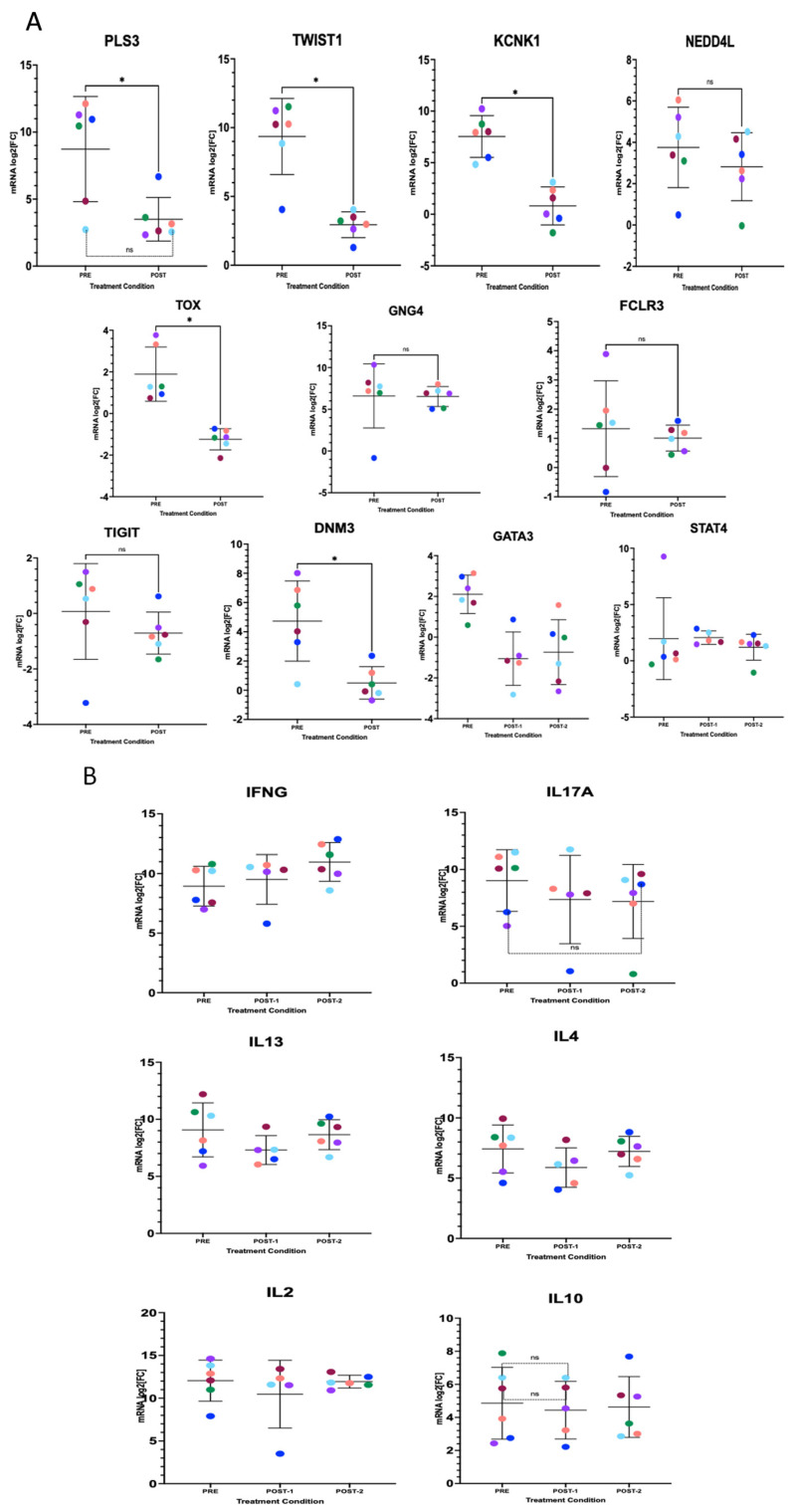
Biomarker gene expression changes with treatment. (**A**) SS biomarker gene expression level in SS PBMCs prior to mogamulizumab therapy (PRE) and initial time postmogamulizumab therapy (POST) was approximately two months. * denotes significance. ns = non-significant. (**B**) Temporal pattern of cytokine gene expression. Cytokine gene expression in SS PBMCs prior to therapy initiation (PRE), approximately two months after therapy (POST1), and approximately one year following therapy (POST2) as measured using qRT-PCR. Each individual denoted by unique color.

After the initial mogamulizumab treatment cycle, all patients showed decreased Sézary cells (CD4^+^CD26^+^ T cells) according to flow cytometry (Table 2). To assess biomarker gene expression level changes with treatment, gene expression from PBMCs was measured at subsequent time points post treatment and compared to gene expression prior to mogamulizumab treatment (Figure 2A) Gene expression analysis using qRT-PCR for SS PBMCs showed that the SS biomarker genes decreased significantly following mogamulizumab treatment for all subjects (Table 3). Overall, *PLS3*, *TWIST1*, *DNM3*, and *KCNK1* showed the largest reduction in expression after treatment (Table 3). The mean level of SS biomarker gene expression after mogamulizumab treatment consistently demonstrated a decrease at two months post mogamulizumab, with the number of genes decreasing to a level comparable to HDs (Table 3).

### 3.4. Defective Cytokine Gene Expression in SS PBMCs and Change After Treatment

We next determined whether there were cytokine gene expression changes in PBMCs of SS patients versus HDs after mogamulizumab treatment. Six cytokine genes reflecting T cell polarization were selected for analysis by qRT-PCR based on prior observations: *IFNG(Th1)*, *IL17A(TH17)*, *IL13(Th2)*, *IL4(Th2)*, *IL2*, and *IL10* [23]. In mogamulizumab-naïve SS subjects, cytokine gene expression in PBMCs showed poor stimulated expression compared to HD PBMCs, consistent with past observations (Figure 2B) [23]. In SS subjects, there was low induction of *IFNG* gene expression compared to HDs and decreased *IL2* gene expression in 5/6 patients. Low induction of cytokine expression was also seen in *IL17A* (2/6), *IL13* (2/6), *IL4* (1/6), and *IL10* (1/6) compared to HDs.

We next determined whether mogamulizumab treatment affects cytokine gene expression in SS PBMCs via qRT-PCR. The average time elapsed following mogamulizumab for gene expression analysis was 61 days in the initial post-treatment specimen and 322 days in the second post-treatment specimen. The variations at time of specimen collection were a consequence of minimizing inconvenience, as this was a real-world study.

The SS PBMC gene expression data for cytokine gene expression after mogamulizumab treatment showed an increase in *IFNG* inducibility and decreased inducible expression of *IL-4* and *IL-13* (Figure 2B). We also measured the expression of transcription factor genes that regulate T cell development, including *GATA3*, which induces Th2 polarization and is increased in SS, and *STAT4*, which induces Th1 polarization and decreases in SS. After mogamulizumab treatment, we observed a decrease in the expression of *GATA3* in all SS subjects, with the lowest level at the second time measurement (Supplementary Table S2). We detected an increase in the expression of *STAT4*, which was most pronounced at the first measurement after mogamulizumab.

### 3.5. Patient Response to Mogamulizumab Treatment

Disease severity was assessed prior to initiation of mogamulizumab and at visits post initial therapy. Subsequent clinical evaluation showed the overall response rate in skin in subjects ranging from PR to CR during the follow up period, with a median duration of 11 months of mogamulizumab therapy. The best response in the skin was CR in two patients and PR in four patients (Table 2). The best response in the blood was PR in five patients. One patient discontinued mogamulizumab therapy due to the development of a rash; nevertheless; this patient showed a significant blood response.

### 3.6. Clinical Response in Skin and Blood to Mogamulizumab Regarding Biomarkers and Cytokines

We next determined the correlation between genes for biomarker expression and clinical response (Table 5). Clinical response scores of the skin and blood compartments were correlated with log2 transformed mean fold-change expression levels of each of the eleven biomarker genes through a linear mixed effect model using JMP version 16.2.0. SS biomarker genes that were significantly correlated with skin response included *PLS3*, *TWIST1*, *KCNK1*, *TOX*, and *DNM3* (*p* < 0.05)—Table 5. Genes that were significantly correlated with blood response included *TOX*, *FCLR3*, and *DNM3*. A subset of SS biomarker genes showed decreased expression after treatment and correlated with the skin response of a decrease in erythema and BSA involvement. A change in skin score was correlated with a decrease in *GATA3*, and a decrease in Sézary cell count was correlated with a negative change in *STAT4* expression. Although we observed a change in cytokine expression after treatment, the change was limited and the correlation to clinical change did not show statistical significance.

## 4. Discussion

In this report, we analyzed the expression of selected highly expressed SS biomarker genes identified from prior transcriptome studies by Moerman-Herzog [8] and genes for Th1 and Th2 polarization from PBMCs from SS patients treated with mogamulizumab in relation to clinical response in a real-world study. There remains a need to incorporate practical biomarkers that are reliably expressed to diagnose and monitor the development and treatment of CTCL. The SS biomarker genes selected in this study were chosen based on their high overexpression compared to normal CD4 T cells from genes identified by Moerman-Herzog [8]. Highly overexpressed novel SS genes that are greater than 10-fold higher than normal offer the advantage of being sensitive biomarker genes that distinguish active SS disease patients from patients with inflammatory skin disease, including genes that can monitor clinical response to treatment with a decreasing burden of malignant cells. T cell immune genes such as T cell transcription factors *GATA3* and *STAT4* are part of the immune gene repertoire and do not show the high-level expression of over 100-fold greater than that of normal T cells, such as that detected for the SS biomarker gene, *PLS3* [5]. Booken et al. also identified differentially expressed genes from Sezary previously, and three of their genes show overlap in our analysis gene set [10]. From the gene expression findings in this report, the data support interrelated conclusions: there are consistent changes in expression patterns in SS biomarker genes compared to normal PBMCs that have diagnostic value, the expression of these biomarker genes responds following mogamulizumab therapy and may correlate with changes in clinical presentation, and mogamulizumab may contribute to immune gene expression restoration. This study demonstrated that monitoring the gene expression of biomarkers in mogamulizumab offers a novel therapeutic assessment for further studies on criteria that are important in defining response to therapy.

Of the genes analyzed, five non-immune biomarker genes were confirmed to be statistically significantly overexpressed in PBMCs from SS compared to non-SS (*PLS3*, *TWIST1*, *KCNK1*, *TOX*, and *DNM3*), consistent with prior results [6,8,9,10]. The uniqueness of *PLS3*, *TWIST1*, *KCNK1* and *DNM3* is that these genes are highly overexpressed in SS compared to HDs, can easily be detected in SS PBMCs, are not normally expressed in immune cells, and thus are effective biomarker genes for monitoring SS. Genes *FCRL3*, *TIGIT*, and *TOX* detected in SS belong to the subset that show consistent elevation in expression in SS compared to non-SS PBMCs, but do not show exceedingly high expression compared to non-immune SS biomarker genes. These genes are not uniquely expressed in SS and are also expressed in Treg and exhausted T cells [12,13,14]. The expression of these T cell genes are indicators of T cell phenotypic shifts in SS, which remain poorly characterized.

The diagnostic potential of the SS biomarker genes was supported by ROC curve analysis. The expression level of *PLS3*, *TWIST1*, *KCNK1*, *TOX*, *DNM3*, and *GATA3* genes demonstrated high specificity (M:97%) and sensitivity (M: 94%) in distinguishing SS from HDs, and the expression level of these genes can be useful in measuring response to treatment (Table 3). Our findings are consistent with prior studies that showed that elevated gene expression of *TWIST1*, *DNM3*, *STAT4*, *GATA3*, and *PLS3* could distinguish 90–98% of SS patients from EID patients [21,25,26]. Thus the results further strengthen the applicability of highly expressed biomarker genes for diagnosing SS and monitoring response to treatment.

After treatment with mogamulizumab, the biomarker genes decreased significantly. However, not all genes reach the baseline-level expression of normal PBMCs. Several SS biomarker genes remained slightly higher in expression than that of normal PBMCs after treatment; however the expression level is significantly lower than that measured prior to treatment. The persistently detectable level of *PLS3* and *TWIST* expression in SS patients after treatment may reflect lingering malignant cells and indicate the sensitivity and the usefulness of these biomarkers for detecting residual disease. There was a significant decrease in Th2 genes, *GATA3,* and Th2 cytokine genes, consistent with a reduction in SS T cells and increased Th1 gene *IFNG* expression from normal T cells, suggesting increased immune function.

Differences in biomarker alterations in patients following treatment may serve as a potential method for assessing the variability in clinical response. Two patients who showed complete response in the skin compartment also demonstrated the largest changes in *TWIST1* and *GNG4* expression and correlation analysis demonstrated that *TWIST1* significantly correlated to skin response. The three patients that showed the largest change in Sézary cell count following therapy also had the largest reduction in *PLS3*, *FCRL3*, *KCNK1*, *TOX*, and *DNM3* expression levels, and *TOX*, *FCRL3*, *DNM3*, and *STAT4* significantly correlated to blood response. Several of the differentially expressed genes that correlated with clinical response were also those with the highest discriminative capacity in SS according to ROC analysis, providing insight into their potential roles in the disease. Although this study used a small cohort, the variability in the preliminary data reveals a significant difference from baseline to after treatment.

The mechanism for mogamulizumab’s clinical response may act via multiple pathways [19,27]. Mogamulizumab acts by targeting neoplastic T cells by binding CCR4 and activating antibody-dependent cellular cytotoxicity. Additional studies have demonstrated that CCR4 expression is not only limited to circulating SS cells but is also expressed on regulatory T cells (Treg) [28,29]. In addition to targeting SS cells, reducing immunosuppressive Treg using mogamulizumab may be important in activating tumor immunity for long-lasting remission. The reduction in Treg by mogamulizumab may be associated with autoimmune skin manifestations in patients with SS treated with mogamulizumab [30,31]. The expression of the immunosuppressive marker, CCR4, on SS T cells may contribute to increased infection and loss of tumor immunity in patients with SS, supporting a role for the use of interferon and photopheresis therapy to stimulate tumor immunity. The decreased expression of *FCRL3* and *TIGIT* after mogamulizumab may be a marker of a reduction in not only tumor cells, but exhausted T cells and Treg cells, which is important in restoring immune balance [32,33,34,35].

Our data demonstrate that the altered expression of highly expressed genes in SS can be directly correlated to the clinical response seen in both the skin and blood. Clinical trials have shown that the effectiveness of mogamulizumab is associated with increased progression-free survival in CTCL, but data on immunotherapeutic assessment remains lacking [21]. In this study, changes in cytokine and T cell transcription factor gene expression that are important in immune functions were observed post treatment. PBMCs from our SS patients prior to treatment showed a Th2 cytokine phenotype with high *GATA3* expression and poor inducibility of *IFNG* and *IL-2* compared to that of normal controls. Post treatment, an increased inducibility of *IFNG* was measured. Developing a Th1 cytokine profile following mogamulizumab is also accompanied by a concomitant decrease in the expression of Th2 genes such as *GATA3*, *IL-4*, and *IL-13*. The reduction in Th2 T cells is significant as the Th2 subset has been linked to the suppression of normal T cells and immune dysregulation [36,37]. In addition, improvement in clinical assessments was associated with decreased *GATA3* and correlated with skin response in SS patients. These cytokines and T cell changes showed that post mogamulizumab treatment, PBMC gene expression shows immune parameters trending to normal.

## 5. Conclusions

Our real-world findings showed that SS biomarkers and cytokine gene expression trend to that of normal following mogamulizumab therapy, supporting the fact that these gene expression changes are markers that are valuable for assessing disease burden and clinical response. This study serves to provide preliminary support for the role of gene expression analysis in monitoring response and supports future comprehensive transcriptomic comparison in a larger cohort, which would eliminate potential bias from the selection. We recognized that a limitation of this study is the small sample size, but the strength of this study is that significant changes in SS biomarker gene expression were readily detected in this patient population. We recognize that other transcriptomic analyses have not identified the same differentially expressed genes that we have reported, and thus we recognize the selection bias in this study. In this SS subset, the findings from the analysis of cytokine genes showed a trend to improvement. Potentially using a larger patient population in a prospective controlled study with a fixed schedule and assessment may yield added clarity regarding the reliability of these SS biomarker genes and the role of T cell transcription factor and cytokine genes in response. In summary, our study demonstrated that changes in the expression of SS biomarker and cytokine genes following treatment may serve as valuable markers for monitoring clinical response in SS patients. Further analysis of larger populations may lead to additional assays to assess the progression or remission of disease.

## Figures and Tables

**Table 1:** Summary of patient characteristics.

Characteristic	SS (*n* = 6)	HDs (*n* = 11)
Male sex, n (%)	4 (67)	9 (82)
Race		
Caucasian	4 (67)	9 (82)
African American	2 (33)	2 (18)
Average age at presentation (years)	65	60
Disease stage, n (%)		
IVA1	4 (67)	−
IVA2	1 (17)	−
IVB	1 (17)	−
Average Sezary cells/uL, median (range)	1045 (13.5–9822)	−
Number of prior therapies, median (range)	5.5 (4–8)	−
Time from diagnosis to mogamulizumab initiation (months), median (range)	15.74 (1.24–116.7)	−
Age at mogamulizumab onset (years), median (range)	67 (41–74)	−
Duration of treatment (months), median (range)	10.50 (6–13)	−
Global overall response rate, n (%)	6 (100)	−
Partial response	6 (100)	−
Complete response	0	−
Skin overall response rate, n (%)	6 (100)	−
Partial response	4 (66)	−
Complete response	2 (33)	−
Blood overall response rate, n (%)	5 (83)	−
Partial response	5 (83)	−
Complete response	0	−

**Table 2:** Clinical response evaluation with treatment.

Patient	Baseline			Post-Treatment 1 (M = 61 days)					Post-Treatment 2 (M = 322 days)				
	BSA	Erythema	Sezary Count	BSA	Erythema	Sezary Count	Skin Response	Blood Response	BSA	Erythema	Sezary Count	Skin Response	Blood Response
1	45%	2	685	60%	2	493	SD	SD	40%	1	82	PR	SD
2	55%	2	510.8	55%	1	56	SD	PR	2%	0.5	49.3	PR	PR
3	80%	1.5	13,777	1%	0.5	35.19	CR	PR	0%	0	42.16	CR	PR
4	85%	4	1239	85%	2	41.9	PR	PR	4%	0.5	57.8	PR	PR
5	75%	1	322	40%	1	24	PR	PR	40%	1	107	PR	PR
6	80%	4	37	60%	2	10.7	PR	PR	0%	0	10.6	CR	PR

Response definition based on mycosis fungoides and Sezary syndrome endpoints [21]: Complete response (CR) is clearance of erythema in the skin. Blood CR is B0. Partial response (PR) in skin is 50–99% decrease in erythema. Blood PR is >50% decrease in Sezary count. Progressive disease (PD) in skin is >25% increase in erythema. PD in blood is >50% increase in Sezary cells. Stable disease (SD) for skin is <25% increase to <50% clearance. SD for blood is not sufficient for CR, PR or PD.

**Table 3:** SS biomarker gene expression and T cell transcription factors compared to HD and SS subjects at baseline and post treatment.

Gene	Healthy Donor	SS: Baseline		SS: Post Treatment	
	M (SD)	M (SD)	*p*-Values	M (SD)	*p*-Values
*PLS3*	4.58 (2.38)	1725.87 (1670.57)	0.0025	23.43 (38.64)	0.0732
*TWIST1*	14.87 (12.40)	1375.33 (1115.80)	0.0018	8.94 (4.76)	0.6928
*KCNK1*	14.79 (8.01)	366.46 (432.88)	0.0040	3.12 (3.21)	0.0099
*NEDD4L*	10.76 (4.85)	23.87 (24.13)	0.1080	10.54 (8.36)	0.8170
*TOX*	0.43 (0.20)	5.34 (5.13)	0.0018	0.44 (0.14)	0.9130
*GNG4*	175.18 (102.77)	345.50 (475.05)	0.3196	118.80 (83.10)	0.7007
*FCLR3*	2.6 (2.19)	4.31 (5.29)	0.3196	2.09 (0.63)	0.8170
*TIGIT*	1.11 (0.80)	1.52 (0.96)	0.1364	0.69 (0.43)	0.3619
*DNM3*	0.83 (0.58)	76.16 (98.66)	0.0292	1.86 (1.70)	0.3160
*GATA3*	0.76 (0.37)	5.03 (2.82)	0.1404	3.81 (2.37)	0.4055
*STAT4*	4.59 (1.72)	103.71 (250.12)	0.0018	0.73 (0.60)	0.3160

**Table 4:** ROC curve analysis for SS biomarker gene performance.

Gene	AUC	*p*-Value	Cut-Off	Sensitivity	Specificity
*TOX*	1	0.0018	>1.242	1	1
*GATA3*	1	0.0018	<1.452	1	1
*PLS3*	0.9848	0.0013	>6.231	1	0.91
*DNM3*	0.9818	0.0027	>1.153	1	0.91
*TWIST1*	0.9697	0.0018	>256.4	0.83	1
*KCNK1*	0.9697	0.0018	>38.17	0.83	1
*STAT4*	0.803	0.0444	>2.065	1	0.67
*NEDD4L*	0.8	0.0616	>8.08	1	0.37
*TIGIT*	0.78	0.0864	>1.284	0.8	0.7
*FCRL3*	0.68	0.2703	>2.578	0.8	0.7
*GNG4*	0.6727	0.2818	>118.8	1	0.37

**Table 5:** Biomarker expression and clinical response correlation to skin and blood response.

Gene	Skin Score Correlation		Sézary Score Correlation	
	R2	*p*-Value	R2	*p*-Value
*PLS3*	0.848	0.02	0.535	0.101
*TWIST1*	0.911	0.004	0.411	0.169
*KCNK1*	0.841	0.015	0.404	0.085
*NEDD4L*	0.583	0.211	0.299	0.271
*TOX*	0.918	0.003	0.421	0.041
*GNG4*	0.517	0.578	0.234	0.219
*FCRL3*	0.498	0.779	0.468	0.012
*TIGIT*	0.576	0.395	0.041	0.231
*DNM3*	0.888	0.006	0.636	0.033
*STAT4*	−0.119	0.351	−1.44	0.004
*GATA3*	0.364	0.033	0.34	0.11

## Data Availability

Data will be provided upon reasonable request.

## Supplementary Materials

The following supporting information can be downloaded at https://www.mdpi.com/article/10.3390/cancers18142304/s1, Table S1: Oligonucleotide primers for PCR of SS biomarker genes; Table S2: Clinical response with treatment.
