# Supplementary material for: Sézary Syndrome Biomarker, T Cell Transcription Factors and Cytokine Genes Provide Novel Insight into Response During Mogamulizumab Treatment
Source: Cancers (Basel). 2026 Jul 17;18(14):2304. doi: 10.3390/cancers18142304 (PMC13406525; doi:10.3390/cancers18142304)
Supplement: Supplementary file 1 [file cancers-18-02304-s001.zip › cancers-4289184-supplementary.pptx]

## Slide 1
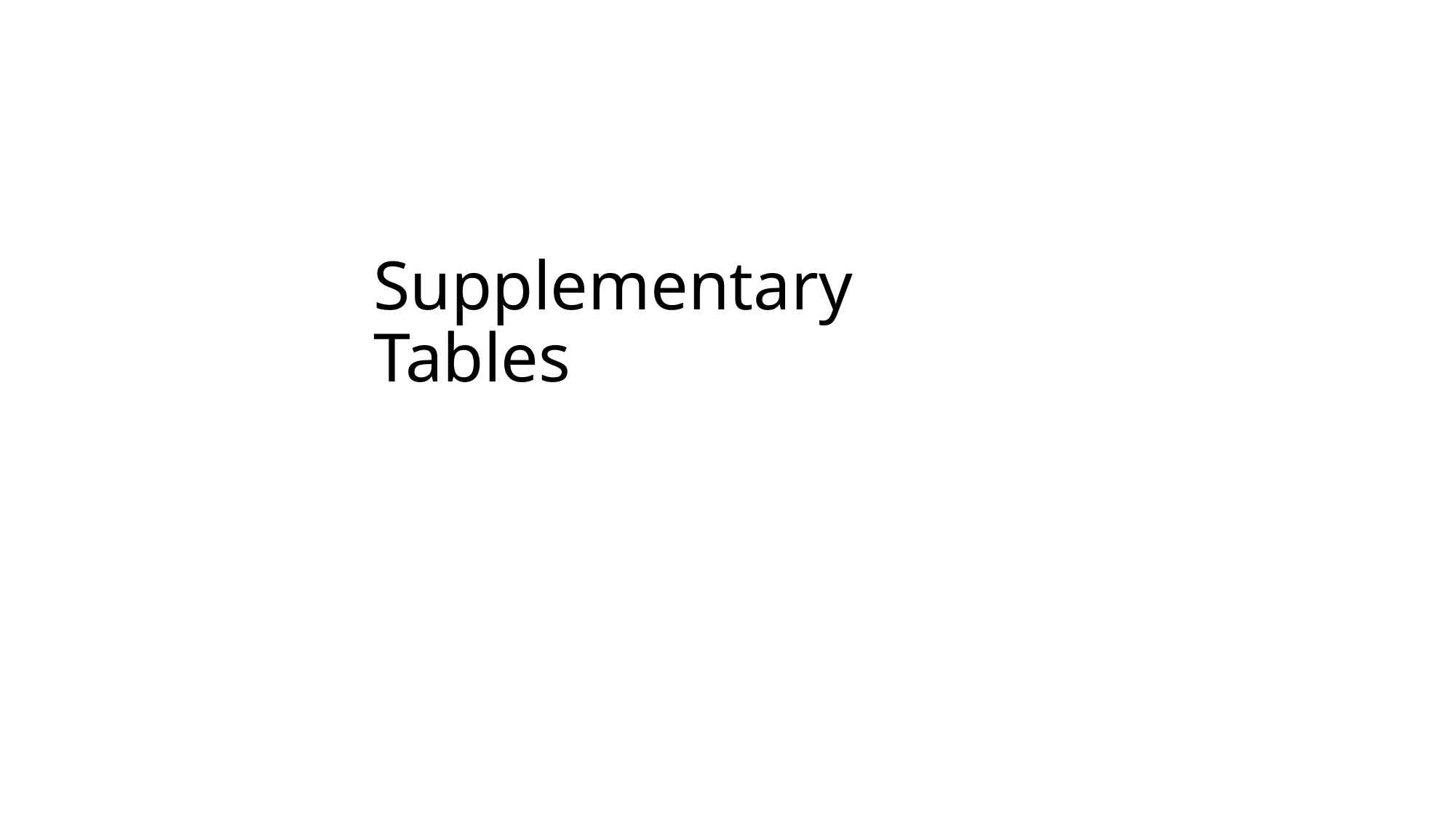

# Supplementary Tables

## Slide 2
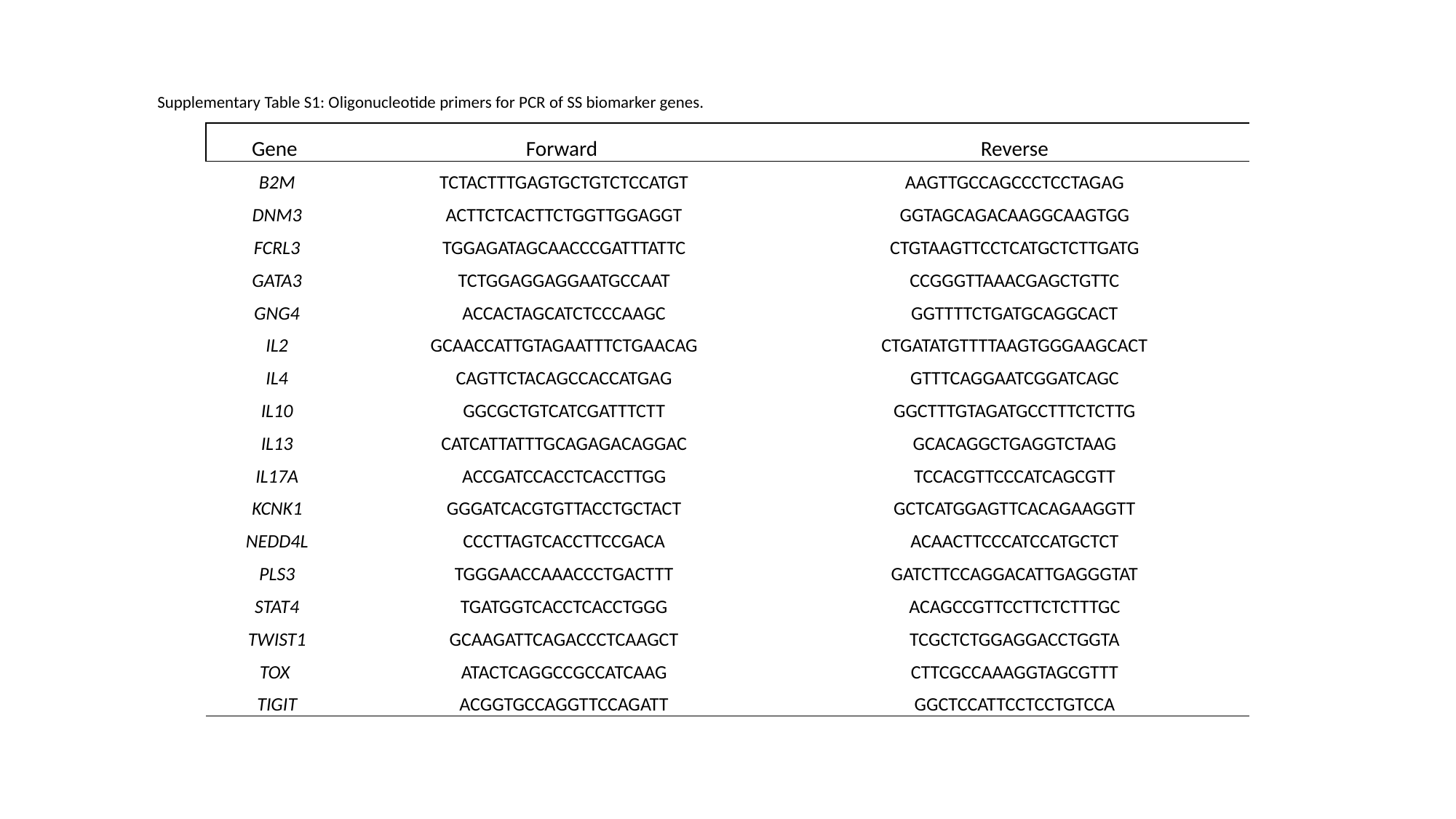

Supplementary Table S1: Oligonucleotide primers for PCR of SS biomarker genes.
| Gene | Forward | Reverse |
| --- | --- | --- |
| B2M | TCTACTTTGAGTGCTGTCTCCATGT | AAGTTGCCAGCCCTCCTAGAG |
| DNM3 | ACTTCTCACTTCTGGTTGGAGGT | GGTAGCAGACAAGGCAAGTGG |
| FCRL3 | TGGAGATAGCAACCCGATTTATTC | CTGTAAGTTCCTCATGCTCTTGATG |
| GATA3 | TCTGGAGGAGGAATGCCAAT | CCGGGTTAAACGAGCTGTTC |
| GNG4 | ACCACTAGCATCTCCCAAGC | GGTTTTCTGATGCAGGCACT |
| IL2 | GCAACCATTGTAGAATTTCTGAACAG | CTGATATGTTTTAAGTGGGAAGCACT |
| IL4 | CAGTTCTACAGCCACCATGAG | GTTTCAGGAATCGGATCAGC |
| IL10 | GGCGCTGTCATCGATTTCTT | GGCTTTGTAGATGCCTTTCTCTTG |
| IL13 | CATCATTATTTGCAGAGACAGGAC | GCACAGGCTGAGGTCTAAG |
| IL17A | ACCGATCCACCTCACCTTGG | TCCACGTTCCCATCAGCGTT |
| KCNK1 | GGGATCACGTGTTACCTGCTACT | GCTCATGGAGTTCACAGAAGGTT |
| NEDD4L | CCCTTAGTCACCTTCCGACA | ACAACTTCCCATCCATGCTCT |
| PLS3 | TGGGAACCAAACCCTGACTTT | GATCTTCCAGGACATTGAGGGTAT |
| STAT4 | TGATGGTCACCTCACCTGGG | ACAGCCGTTCCTTCTCTTTGC |
| TWIST1 | GCAAGATTCAGACCCTCAAGCT | TCGCTCTGGAGGACCTGGTA |
| TOX | ATACTCAGGCCGCCATCAAG | CTTCGCCAAAGGTAGCGTTT |
| TIGIT | ACGGTGCCAGGTTCCAGATT | GGCTCCATTCCTCCTGTCCA |

## Slide 3
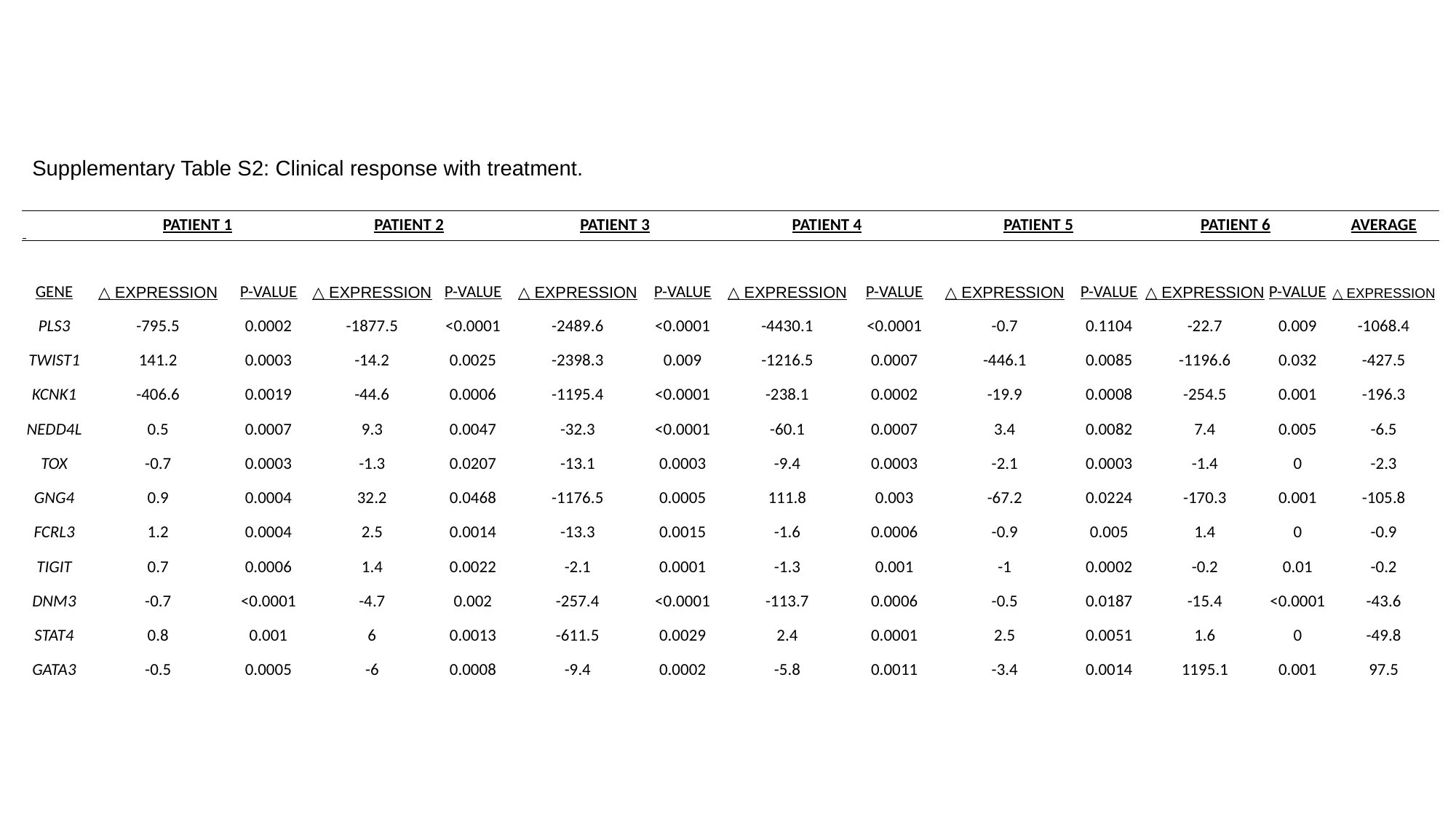

Supplementary Table S2: Clinical response with treatment.
| | PATIENT 1 | | PATIENT 2 | | PATIENT 3 | | PATIENT 4 | | PATIENT 5 | | PATIENT 6 | | AVERAGE |
| --- | --- | --- | --- | --- | --- | --- | --- | --- | --- | --- | --- | --- | --- |
| GENE | △ EXPRESSION | P-VALUE | △ EXPRESSION | P-VALUE | △ EXPRESSION | P-VALUE | △ EXPRESSION | P-VALUE | △ EXPRESSION | P-VALUE | △ EXPRESSION | P-VALUE | △ EXPRESSION |
| PLS3 | -795.5 | 0.0002 | -1877.5 | <0.0001 | -2489.6 | <0.0001 | -4430.1 | <0.0001 | -0.7 | 0.1104 | -22.7 | 0.009 | -1068.4 |
| TWIST1 | 141.2 | 0.0003 | -14.2 | 0.0025 | -2398.3 | 0.009 | -1216.5 | 0.0007 | -446.1 | 0.0085 | -1196.6 | 0.032 | -427.5 |
| KCNK1 | -406.6 | 0.0019 | -44.6 | 0.0006 | -1195.4 | <0.0001 | -238.1 | 0.0002 | -19.9 | 0.0008 | -254.5 | 0.001 | -196.3 |
| NEDD4L | 0.5 | 0.0007 | 9.3 | 0.0047 | -32.3 | <0.0001 | -60.1 | 0.0007 | 3.4 | 0.0082 | 7.4 | 0.005 | -6.5 |
| TOX | -0.7 | 0.0003 | -1.3 | 0.0207 | -13.1 | 0.0003 | -9.4 | 0.0003 | -2.1 | 0.0003 | -1.4 | 0 | -2.3 |
| GNG4 | 0.9 | 0.0004 | 32.2 | 0.0468 | -1176.5 | 0.0005 | 111.8 | 0.003 | -67.2 | 0.0224 | -170.3 | 0.001 | -105.8 |
| FCRL3 | 1.2 | 0.0004 | 2.5 | 0.0014 | -13.3 | 0.0015 | -1.6 | 0.0006 | -0.9 | 0.005 | 1.4 | 0 | -0.9 |
| TIGIT | 0.7 | 0.0006 | 1.4 | 0.0022 | -2.1 | 0.0001 | -1.3 | 0.001 | -1 | 0.0002 | -0.2 | 0.01 | -0.2 |
| DNM3 | -0.7 | <0.0001 | -4.7 | 0.002 | -257.4 | <0.0001 | -113.7 | 0.0006 | -0.5 | 0.0187 | -15.4 | <0.0001 | -43.6 |
| STAT4 | 0.8 | 0.001 | 6 | 0.0013 | -611.5 | 0.0029 | 2.4 | 0.0001 | 2.5 | 0.0051 | 1.6 | 0 | -49.8 |
| GATA3 | -0.5 | 0.0005 | -6 | 0.0008 | -9.4 | 0.0002 | -5.8 | 0.0011 | -3.4 | 0.0014 | 1195.1 | 0.001 | 97.5 |
